# Supplementary material for: The mutational signatures of formalin fixation on the human genome
Source: Nat Commun. 2022 Sep 6;13:4487. doi: 10.1038/s41467-022-32041-5 (PMC9448750; doi:10.1038/s41467-022-32041-5)
Supplement: Supplementary file 1 — Supplementary Information [file 41467_2022_32041_MOESM1_ESM.pdf]

Supplementary Information

The mutational signatures of formalin fixation on the human genome

Qingli Guo, Eszter Lakatos, Ibrahim Al Bakir, Kit Curtius, Trevor A. Graham, Ville Mustonen

Supplementary Table 1 FFPE artefacts in other publications

Studies	Sample information	DNA extraction kit	Types of NGS	Sequencing platform	UDG	Predominate artefacts	Authors' results/conclusions
Flores Bueso *et al*.[1]	Escherichia coli cells	QIAGEN FFPE DNA kit	WGS	Illumina HiSeq	Yes & No	C>T	"Our data suggest that DNA damage found in bacterial FFPE DNA is primarily driven by oxidation and cytosine deamination"
Chen *et al*.[2]	FFPE samples (n=7)	Ion AmpliSeq Library Kit 2.0	TES	Personal Genome Machine (PGM) sequencing platform	Yes & No	C>T	"The baseline noise in normal peripheral blood and formalin-fixed paraffin-embedded samples detected by next-generation sequencing (NGS) is dominated by C:G>T:A mutations, which are signature mutations of cytosine deamination"
Williams *et al.*[3]	basal cell cancer (n=1)	NA	TES	solid-phase sequencing	Not found	C>T	"A total of 28 artificial mutations were recorded, of which 27 were C-T or G-A transitions. "
Do *et al.*[4]	Squamous cell lung-carcinomas (n=3)	DNeasy Tissue and Blood KIT(Qiagen)	TES	Illumina MiSeq	Yes & No	C>T	"When the prevalence of each SNC type was examined, C:G>T:A were by far the most frequent in all 3 samples"
Do & Dobrovic *et al*.[5]	Squamous cell lung carcinomas(n=5)	DNeasy Tissue and Blood kit (Qiagen)	TES	Sanger Sequencing	Yes & No	C>T	"the sequence artefacts detected in the FFPE tumour DNAs were almost exclusively C:G>T:A base substitutions (16/17)"; "Sequencing of these samples showed multiple non-reproducible C:G>T:A artefacts"
Yost *et al.*[6]	Breast cancers (n=2)	BiOstic FFPE Tissue DNA Isolation kit (MO BIO, Carlsbad, CA, USA)	WGS	SOLID	Not found	C>T	"The tumor samples show differing amounts of FFPE damaged DNA sequencing reads revealed as relatively high alignment mismatch rates enriched for C · G > T · A substitutions compared to germline samples"
Spencer *et al.*[7]	lung adenocarcinoma (n=16)	QIAmp Micro DNA kit (Qiagen, Valencia, CA)	TES	Illumina HiSeq 2000	Not found	C>T	"C to T transitions were significantly increased in FFPE tissue compared with frozen tissue ($P = 3.98 \times 10^{-10}$, Student's *t* -test), with a corresponding increase in G to A transitions"

Oh *et al.*[8]	Cancer sample (n=5)	unknown	WES	Illumina HiSeq 2000	Not found	C>T	"In this analysis, we re-confirmed that C>T and G>A base transitions occurred specifically in the FFPE samples as formalin fixation artifacts."
Serizawa *et al.* [9]	esophageal cancer(n=135)	QIAamp DNA FFPE tissue kit (Qiagen, Venlo, Netherlands)	TES	MiSeq sequencer (Illumina)	Yes & No	C>T	"We also confirmed the efficacy of UDG pretreatment in reducing C:G > T:A SNVs, which are the predominant type of sequencing artifacts observed in FFPE DNA"
Lin *et al.*[10]	Lymph node tissues (n=16), neoplastic tissues (n=118)	QIAmp DNA kit (Qiagen, Valencia, CA)	TES	Pyrosequencing and sanger sequencing	No	C>T	"Baseline noise is consistent with spontaneous and FFPE-induced C:G -> T:A deamination mutations "
Ofner *et al.*[11]	Melanoma (n=96)	Biostic FFPE Tissue DNA Isolation-kit	TES	Sanger Sequencing	Not found	C>T	"C>T: 77.1%" (Table 2);<br>"In our case, 8 out of 11 non-reproducible artifacts were C:G>T:A transitions"
Gallegos Ruiz *et al.*[12]	non-small cell lung cancer(n=47)	QIAamp DNA mini kit	TES	Sanger Sequencing	No	C>T	"As detailed in Table 2, all artifactual mutations resulted from C>T or G>A transitions"
Sah *et al*.[13]	Cancer samples (n=44)	RecoverAll™ Total Nucleic Acid Isolation Kit for FFPE (Life Technologies)	TES	MiSeq (Illumina, San Diego, CA, USA)	No	C>T	"we observed that 75% of the false positives reported in Table 1 were C>T or G>A transition mutations."
Alborelli *et al.*[14]	Lung carcinoma (n=12)	Maxwell® 16 FFPE Plus LEV DNA kit (Promega, Wisconsin, USA, Cat.No. AS1135)	TES	Ion S5XL™ instrument (Thermo Fisher Scientific).	Yes	C>T	"Discordant variants were mainly unique to FFPE samples (34/40 discordant variants) and mostly C:G > T:A transitions with low allelic frequency, likely indicating formalin fixation artifacts"

Parker *et al.*[15]	Colon cancer (n=10)	FFPE DNA-extraction kits (Qiagen, Toronto, ON, Canada)	WES	HiSeq 2500 instruments (Illumina)	Not found	C>T	"The excess variants were classified as being consistent with the result of a base transition event due to cytosine deamination, which we refer to here as an FFPE transition variant (ie, C>T or G>A transitions)."
Quach *et al.*[16]	Normal colon	DNeasy Tissue Kit, Qiagen, Valencia, CA	TES	ABI 377XL automated sequencer	No	C>T & T>C	"Mutation types were different after fixation, with a predominance (92%) of transition mutations"; "Point mutations at A:T base pairs were significantly (p= 0.034) more frequent than at G:C pairs in the fixed DNA (2.9 to 1 versus a ratio of 1.2 to 1 in this DNA sequence)"
Marchetti *et al.*[17]	Lung-tumor (n=70)	phenol–chloroform protocol	TES	Sanger sequencing	No	C>T & T>C	"22 (92 percent) of these mutations were C→T/G→A or A→G/T→C transitions." ; "All the uncommon mutations detected were found to be artifacts. "
Wong *et al.*[18]	Ovarian cancer (n=70)	Not found	TES	Sanger sequencing	No	C>T & T>C	"There were 42 transitions (76%) and 13 transversions (24%). Transitions included 23 GC>AT and 19 AT>GC. Transversions included 7 GC>TA and 6 AT>TA."
Do & Dobrovic *et al.*[19]	non-small cell lung cancer(n=4)	QIAamp DNA blood kit (Qiagen, Hilden, Germany)	TES	Sanger Sequencing	No	C>T	"In this study, nearly all the base changes were G to A or C to T mutations"
	non-small cell lung cancer(n=1)	QIAamp DNA blood kit (Qiagen, Hilden, Germany)	TES	Sanger Sequencing	No	C>T & C>A	8/17 C>T; 9/17 C>A, according to Table 2 (HotStar HiFidelity, Qiagen)
Lamy *et al.*[20]	Colorectal cancer (n=1,130)	RecoverAll™ Total Nucleic Acid Isolation Kit	TES	ABI PRISM 3130*xl* Genetic Analyzer	Yes	C>T & C>A	"As a whole, 283 *KRAS* artefactual mutations were recorded from 187 analyses: 148 (52.3%) corresponded to G>A transitions, 103 (36.4%) to G>T transversions, and 32 (11.3%) were G>C transversions."

n: FFPE sample size; TES: targeted exon sequencing; WES: whole exome sequencing; WGS: whole genome sequence

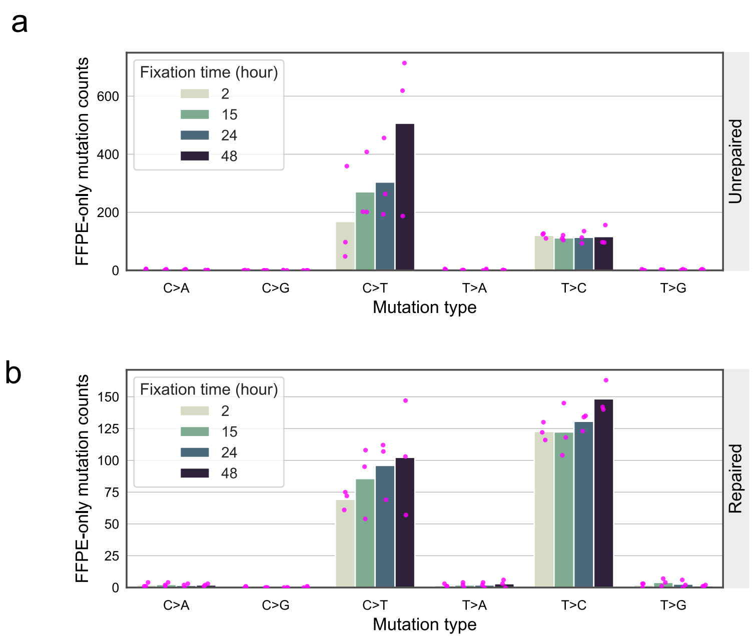

Supplementary Fig. 1 FFPE-only mutations with increasing formalin fixation time. FFPE-only mutations here refer to those that are not present in FF sample and known germline databases. The data is taken from the fixation group in study 1[21] (see Methods). (see Methods). (**a-b**) Mutation count for six mutation types in unrepaired (**a**) and repaired (**b**) FFPE samples. For each mutation type, we show the mutation counts detected in four FFPE samples being fixed in formalin for 2, 15, 24 and 48 hours, respectively. The bar height represents the mean of FFPE only mutation count, and the individual count in *n*=3 patients is marked as pink dots.

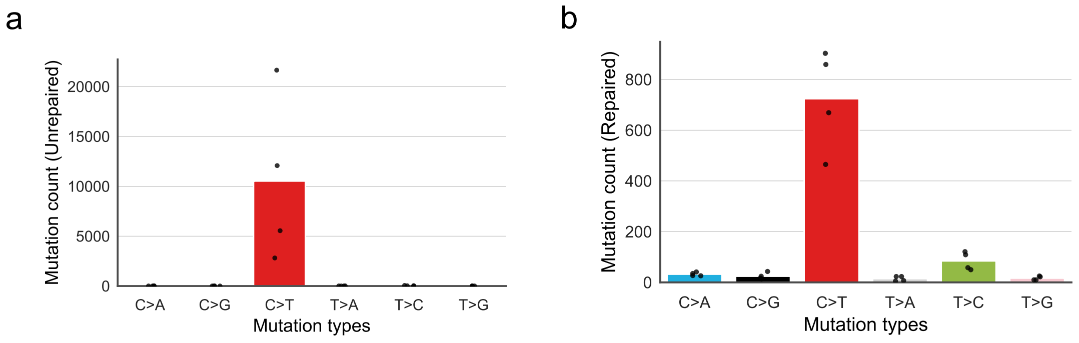

Supplementary Fig. 2 FFPE-only mutations in six basic mutation types in study 2[22]. FFPE-only mutations here refer to those that are not present in FF samples and known germline databases. **a-b**, Mutation counts of six basic mutation types for FFPE-only mutations in study 2 for unrepaired FFPEs (**a**) and for repaired FFPEs (**b**). The bar height represents the mean of FFPE only mutation count, and the individual count in *n*=4 individuals is marked as black dots.

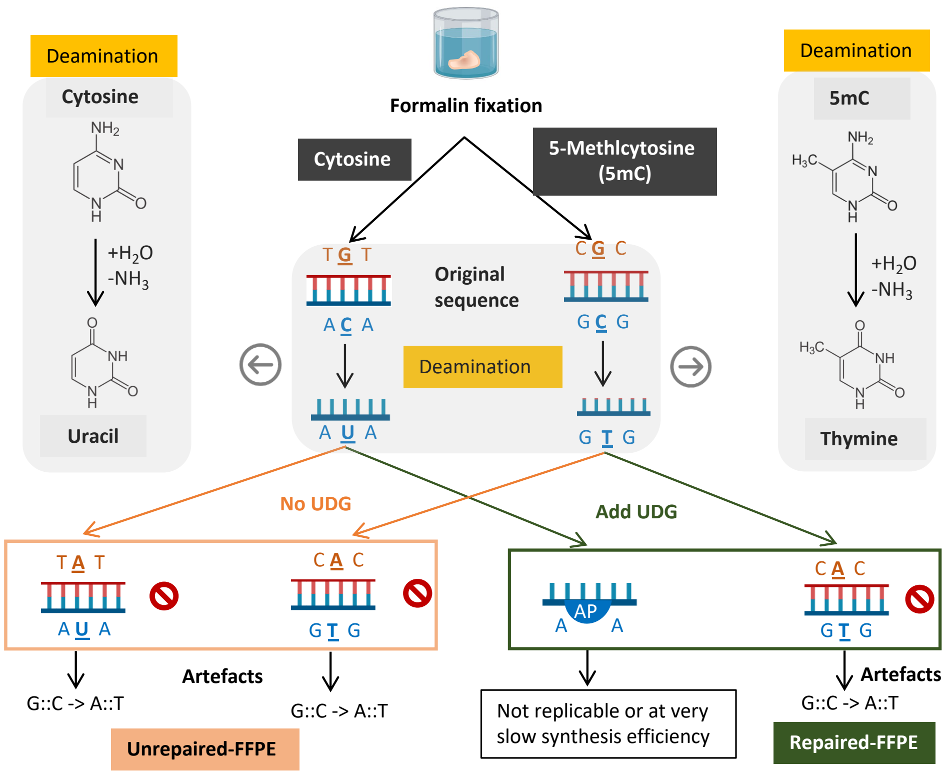

Supplementary Fig. 3 The hydrolytic deamination of cytosine and 5-methylcytosine lead to C>T/G>A artefacts in FFPE samples via different pathways. Formalin fixation could cause hydrolytic deamination of cytosine bases to uracil (left grey-panel), and the deamination of 5-methylcytosine (5mC) in CpG dinucleotides converts directly to thymine (right grey-panel). The deamination results in U:G mismatches where DNA polymerase incorporates adenine opposite to uracil in amplicon-based protocols, generating artefactual C:G>T:A substitutions in sequencing data. To mitigate deamination artefacts, some FFPE sequencing library preparations include repair treatment whereby uracil DNA glycosylase (UDG) is added to remove uracil bases prior to amplification. This repair method removes uracil but leaves the abasic sites (AP sites) on the DNA templates, which are typically not replicable or at very slow synthesis efficiency. Therefore, only a small number of artefacts would appear in the sequence data. However, UDG does not repair artefacts pathways from 5mC. Parts of the figure were drawn by using pictures from Servier Medical Art. Servier, which is licensed under a Creative Commons Attribution 3.0 Unported License (https://creativecommons.org/licenses/by/3.0/).

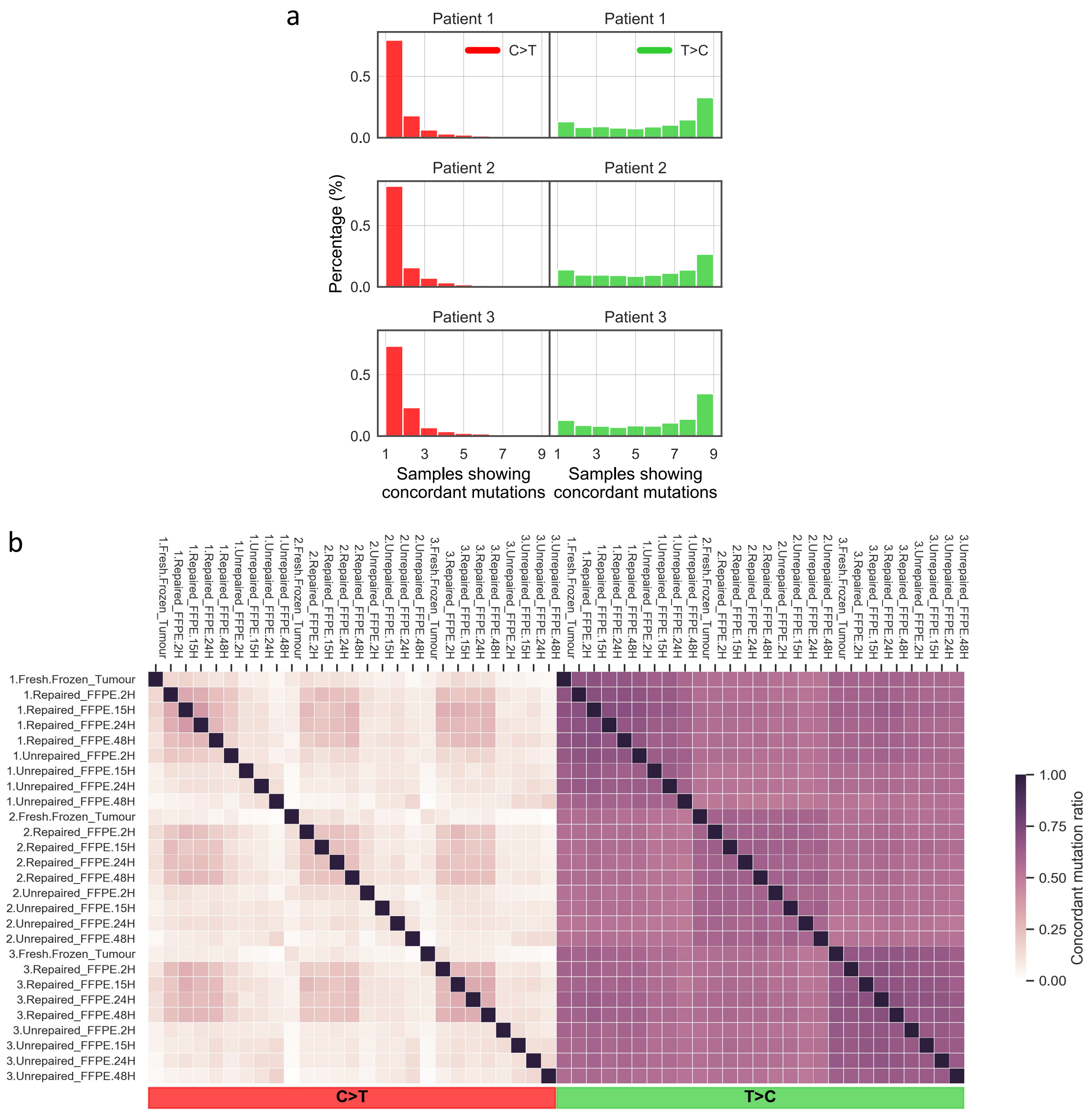

Supplementary Fig. 4 T>C mutations are highly repeated among samples. a, Normalised histogram of concordant mutation count per patient. We used raw primary mutation candidates of the fixation group (*n*=27) from study 1 (FF samples are available). This primary list contains FFPE artefacts, unfiltered SNPs as well as system errors and true somatic mutations. We take all T>C and C>T mutations from the whole mutation list and count the occurrences the mutations among all *n*=9 samples (4 repaired FFPEs, 4 unrepaired FFPEs and 1 FF) for each patient. **b**, Pair-wise concordant mutation ratios for *n*=27 samples from three patients. Concordant mutation ratio is calculated using concordant mutation numbers within a sample pair divided by their total unique mutation count.

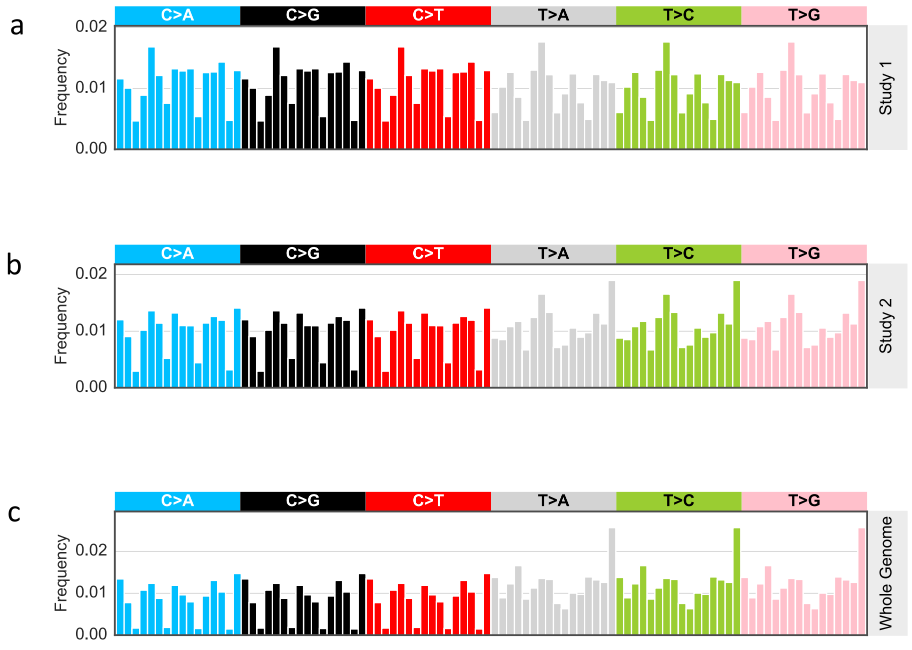

Supplementary Fig. 5 Mutational opportunities. a-c, Mutational opportunities for study 1 (**a**), for study 2 (**b**) and for whole genome sequence context (**c**).

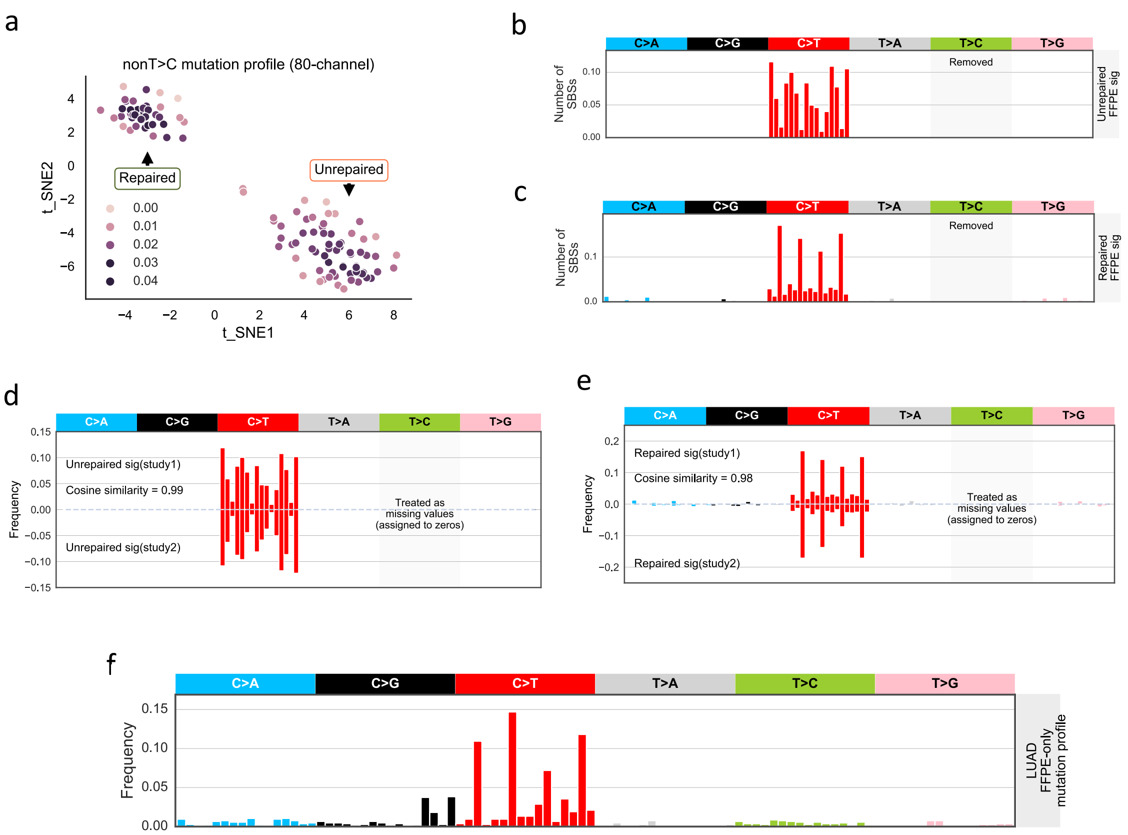

Supplementary Fig. 6 Deriving and validating FFPE signatures. a, Scatter plot of spatial density of t-SNE clustered samples. The density is measured using gaussian kernel. Samples with top 50% density value were used for deriving FFPE signatures using the averaged mutational channels. **b-c**, Derived unrepaired (**b**) and repaired (**c**) FFPE signature. We repeated (**a**) for 100 times using different random seeds. The final version of the unrepaired FFPE signature takes the averaged values of all 100 candidates. **d-e**, Almost identical unrepaired (**d**) and repaired (**e**) FFPE signatures generated from independent analysis using real FFPE samples from study 1 and 2. We applied the same methodology described in our Methods for deriving FFPE signatures from study 1 (*n*=102 FFPE samples). We took the averaged mutation profiles from study 2 to obtain the signatures (*n*=8 FFPE samples). The two sets of signatures are highly similar to each other. **f,** The aggregated FFPE-only mutation profile from *n*=11 lung adenocarcinoma (LUAD) samples. FFPE only mutations are found in the filtered mutation list of FFPE samples in study 3 by Van Allen *et al.*[23]. It shares a highly similar pattern with our discovered repaired FFPE signature (cosine similarity = 0.93).

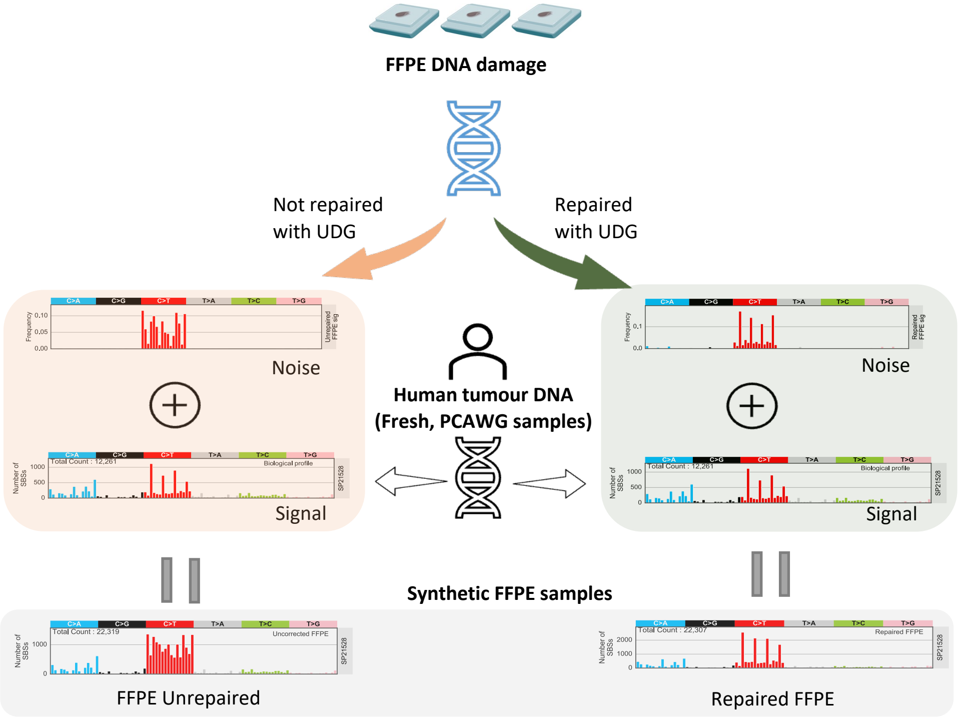

Supplementary Fig. 7 The process of generating synthetic FFPE samples. To simulate FFPE samples, we mixed the FFPE noise to the biological mutation profiles derived from fresh frozen tumours in the PCAWG project[24,25]. In the main simulation set-up, the FFPE noise count is about 10^4. The unrepaired FFPE artefacts are generated from the unrepaired signature (Supplementary Fig. 6b) and repaired FFPE artefacts are generated from the repaired signature (Supplementary Fig. 6c). Note that we omitted T>C mutations in all our final synthetic FFPE samples. Parts of the figure were drawn by using pictures from Servier Medical Art. Servier, which is licensed under a Creative Commons Attribution 3.0 Unported License (https://creativecommons.org/licenses/by/3.0/).

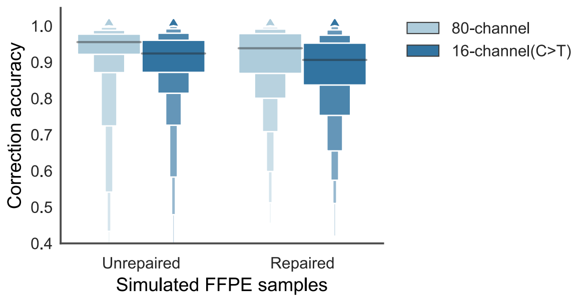

Supplementary Fig. 8 Comparison of correction accuracy of FFPEsig. We measured the correction accuracy on corrected *n*=2,780 synthetic FFPE samples using either 80-channel (without T>C) or on 16-channel C>T channels, where the dominant formalin-induced artefacts distributed. We applied the stricter accuracy measure on C>T channels in our study. All data are independent and presented using a Letter-Value plot and the black line in the middle box corresponds to the median of the dataset. Every further step splits the remaining data further into two halves.

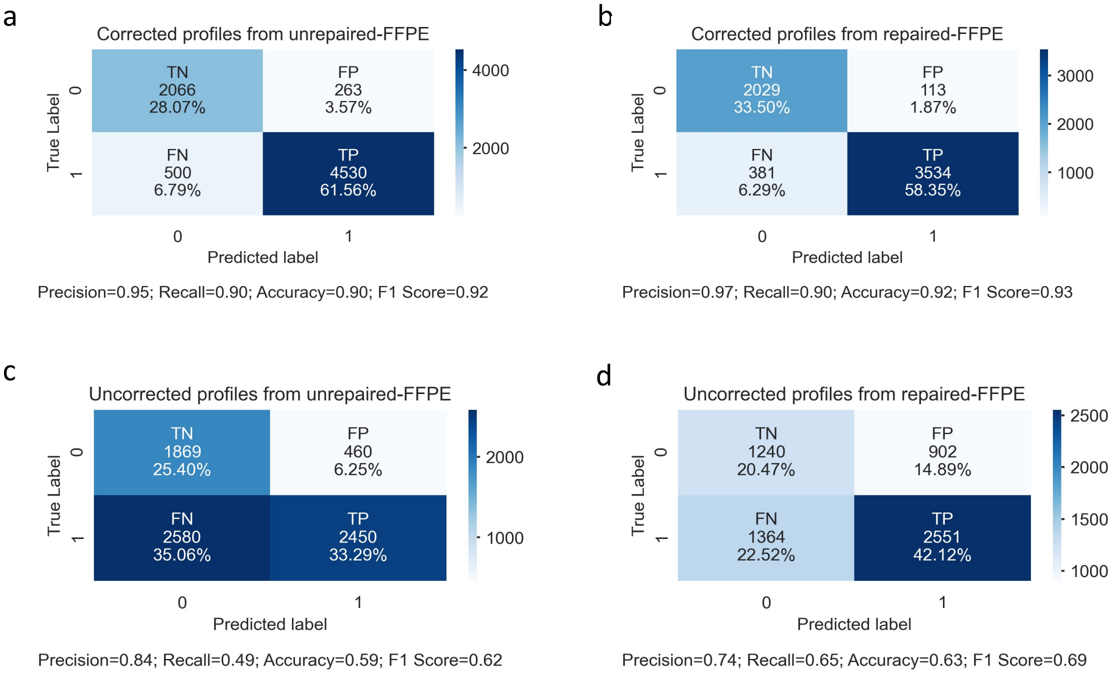

Supplementary Fig. 9 Confusion matrix of binary classification of assigned activities. a-b, Binary classification results using corrected profiles by FFPEsig. We assigned label 1 to signatures with activity weights of 0.1 or more (being present in the given tumour), and 0 to signatures with contribution smaller than 0.1 (being absent). The true labels (y-axis) are determined using activities inferred from true biological profiles. And the predicted labels are determined using weights inferred from simulated unrepaired (**a**) and repaired (**b**) FFPE samples. TN: true negatives; TP: true positives; FP: false positives; FN: false negatives. Precision = TP/(TP+FP); recall = TP/(FN+TP); accuracy = (TN+TP)/(TN+TP+FN+FP); F1 Score = 2 * precision * recall / (precision+recall). **c-d,** Binary classification results using uncorrected profiles for unrepaired (**c**) and for repaired (**d**) FFPEs.

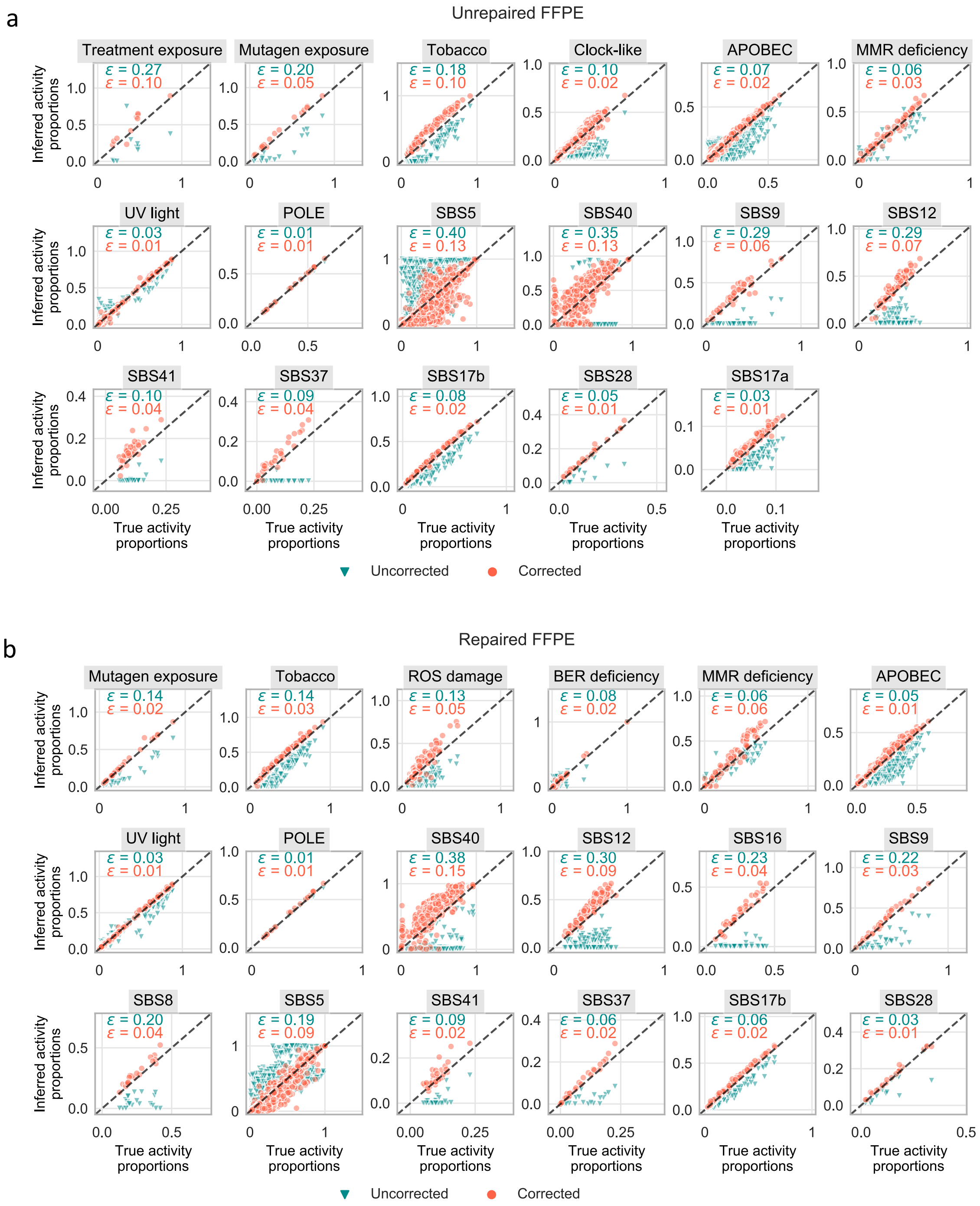

Supplementary Fig. 10 Comparison of signature activities decomposed from corrected and uncorrected FFPE profiles. a-b, Comparison of activity proportions inferred using corrected or uncorrected FFPE profile against true activity proportions for unrepaired (**a**) and repaired (**b**) FFPE. The true activity proportions are inferred using the biological profiles. Mean of the errors (ε) for each comparison is also included (upper left) with the same colour of the data where it is derived from.

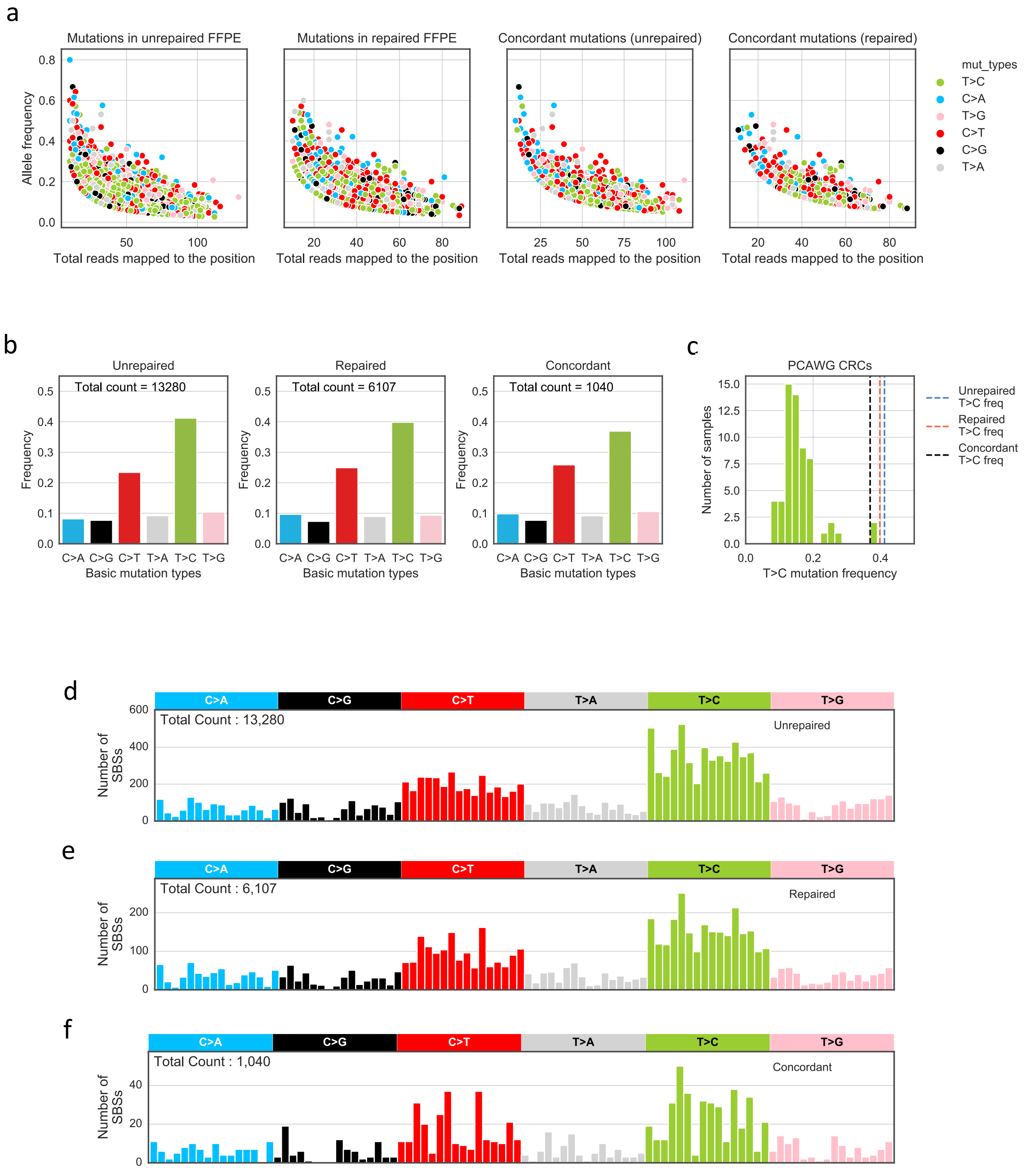

Supplementary Fig. 11 Observed mutations from two WGS FFPE CRC samples. a. Allele frequency versus total reads number (REF+ALT) for all mutations. The four panels from left to right show mutations detected from unrepaired FFPE, repaired FFPE and concordant mutations in unrepaired and concordant mutations in repaired FFPEs, respectively. Concordant mutations refer to variants that are detected in both repaired and unrepaired FFPEs with at least 5 supporting reads. **b,** Total count of SBS variants in unrepaired, repaired and concordant mutations. **c,** T>C mutation frequencies of PCAWG CRC samples. Three dash lines indicate T>C mutation frequencies of unrepaired, repaired and concordant mutations from our sequenced FFPE samples. **d-f**, The 96-channel mutation profiles of unrepaired (**d**), repaired (**e**) and concordant mutations present in both unrepaired and repaired CRC samples (**f**).

a

b

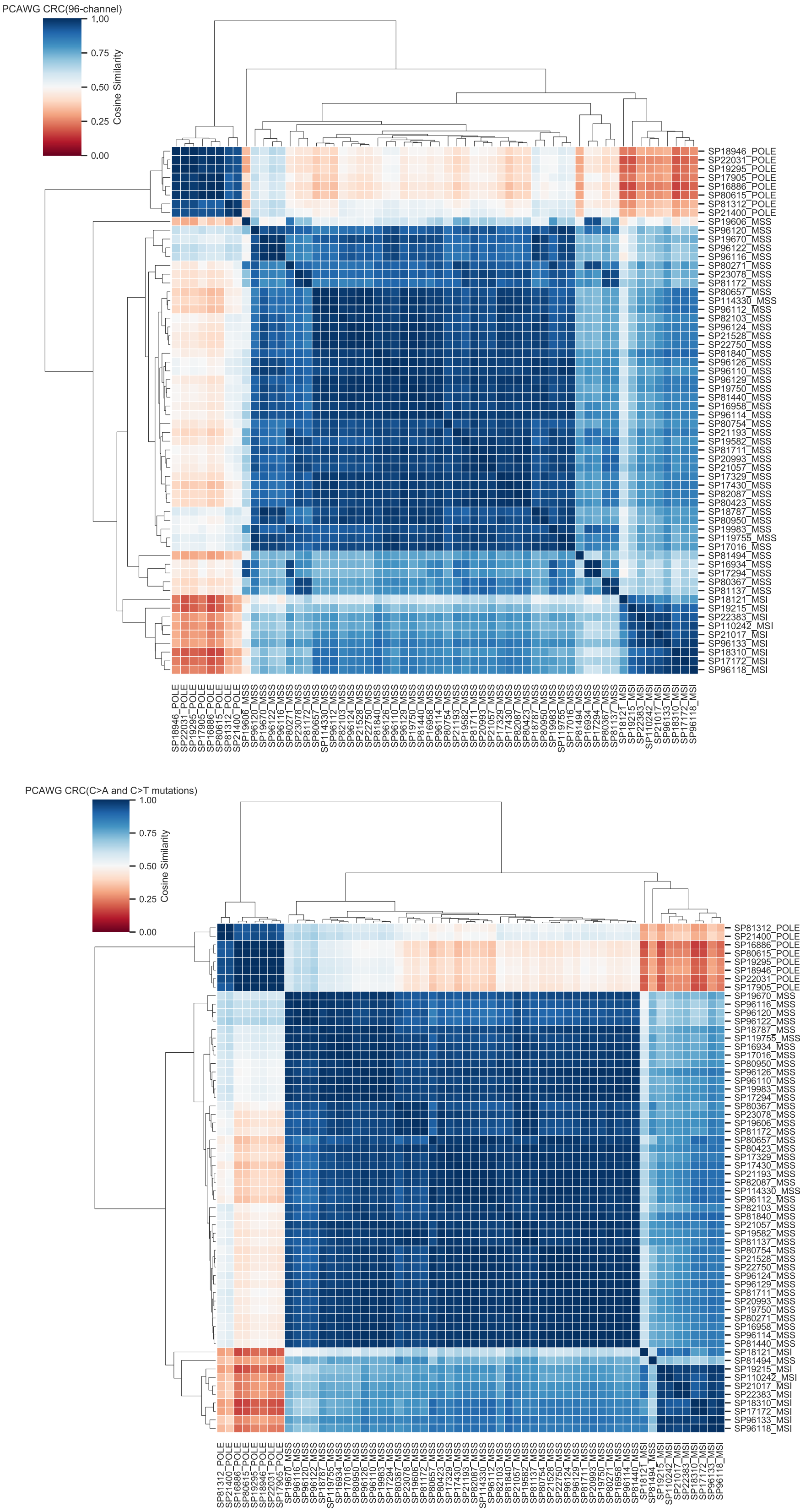

Supplementary Fig. 12 Cluster of PCAWG CRCs using mutational catalogues. a, using full 96-channel profiles. **b,** using C>A and C>T mutation profiles (32 channel).

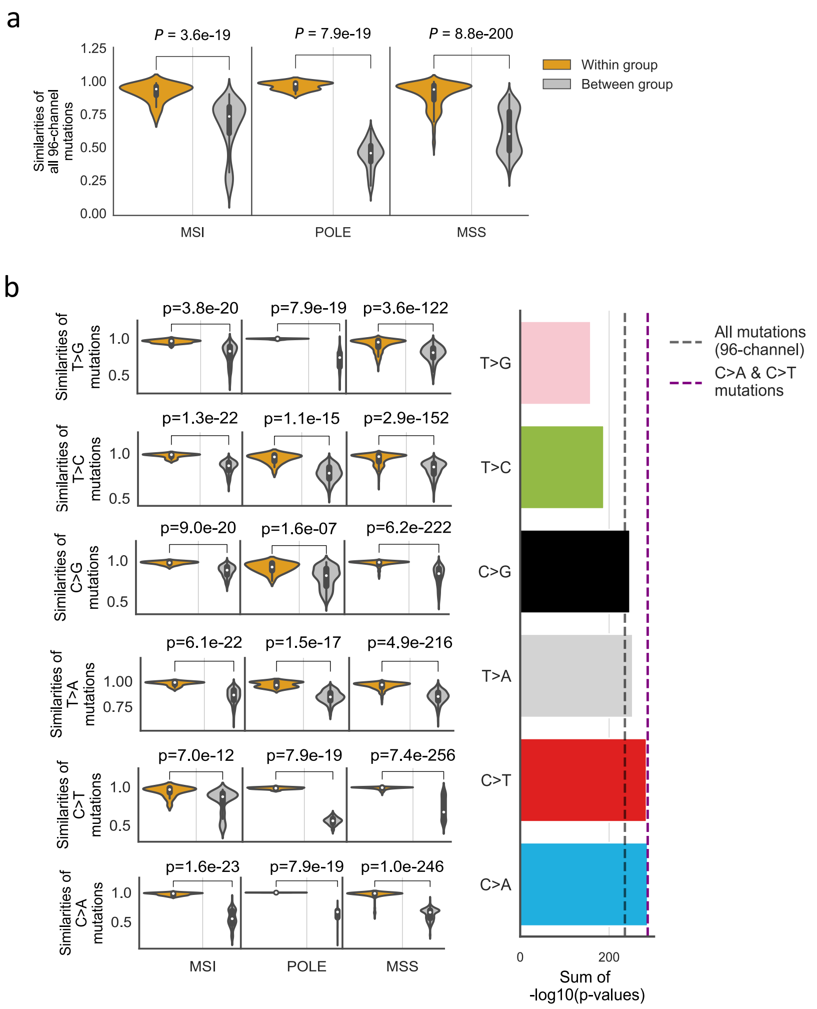

Supplementary Fig. 13 Comparison of sample-pair similarities within and between subgroups of PCAWG CRCs. a-b, Sample-pair similarities of full 96-channel (**a**) or 16-channels of one basic mutation type (**b**). We calculated the cosine similarities of sample pairs within and between three subgroups (POLE, MSI and MSS). In total, we obtained *n*=28 (POLE-POLE), *n*=36 (MSI-MSI), and *n*=903 (MSS-MSS) independent sample pairs within three subgroups (orange colour), and *n*=416 (POLE-MSS/MSI), *n*=459 (MSI-POLE/MSS) and *n*=731 (MSS-MSI/POLE) independent sample pairs between the given subgroup and the other two subgroups. CRC: colorectal cancer; POLE: polymerase epsilon mutated; MSS: microsatellite stable; MSI: microsatellite instability. Data are presented using violin plot. The white dot represents the median. The thick grey bar in the center represents the interquartile range and the thin grey line represents the rest of the distribution. The *P*-values of differences for each subgroup are shown above each box-pair using the two-sided Mann-Whitney *U* test. We use the sum of -$\log_{10}$ (*p*-value) to sort the six mutation types, shown in the right panel. We also use black and purple dash lines to mark sums of -$\log_{10}$ (*P*-value) values by using 96-channel and by using C>A and C>T (32-channel).

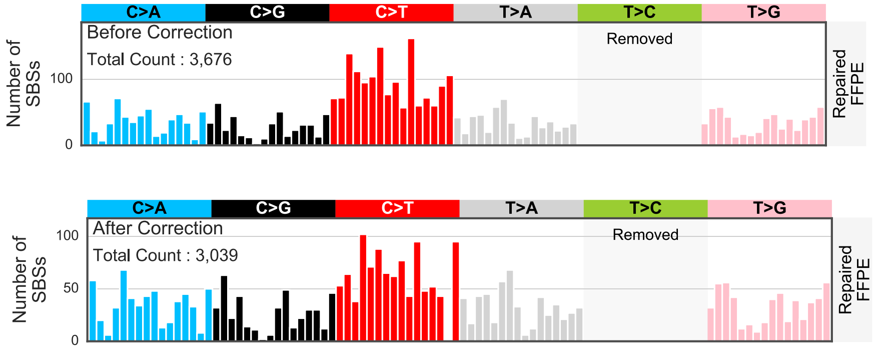

Supplementary Fig. 14 FFPE noise correction results of repaired FFPE CRC sample. The top panel shows the mutational profile before correction. And the lower panel shows the corrected profile. We here removed the T>C mutations for a clearer visualisation of correction results on C>T channels. The full 96-channel of profile can be found in Supplementary Fig. 11.

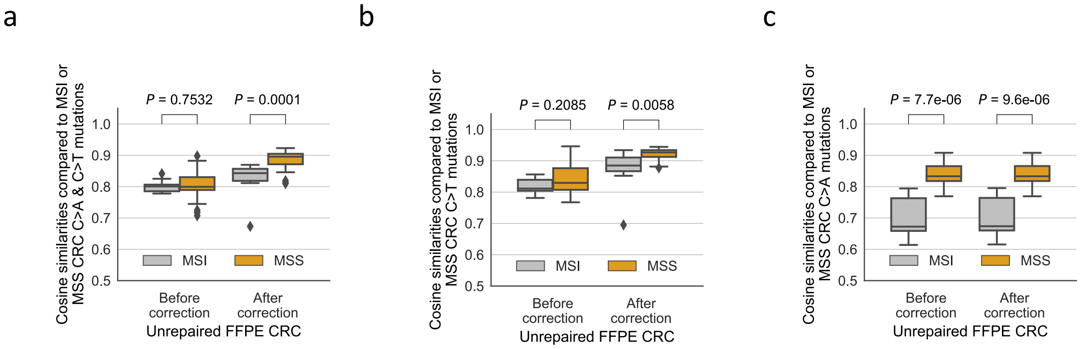

Supplementary Fig. 15 Correction on unrepaired FFPE CRC sample contributes to classify MSS from MSI subtype. a-b, Significant accuracy for the classification using corrected profiles by FFPEsig when using C>A and C>T mutations (**a**) and C>T mutation pattern only (**b**). The corrected and uncorrected profiles were compared to *n*=9 MSI-CRC and *n*=43 MSI-CRC samples. The significant difference for each subgroup is measured by two-sided Mann-Whitney *U* test on independent data points. The black line in each box marks the median of the data, and the upper and lower bounds of the box indicate the inter-quantile range. The upper and lower whiskers represent scores outside the middle 50%. **c**, Not significant improvement for the subtype classification using C>A profiles alone. As our correction acts on C>T channels mostly, so the C>A mutation remained almost the same before and after correction (cosine similarity= ~0.99), and their profile maintained as the most conserved pattern (Supplementary Fig. 13b) .

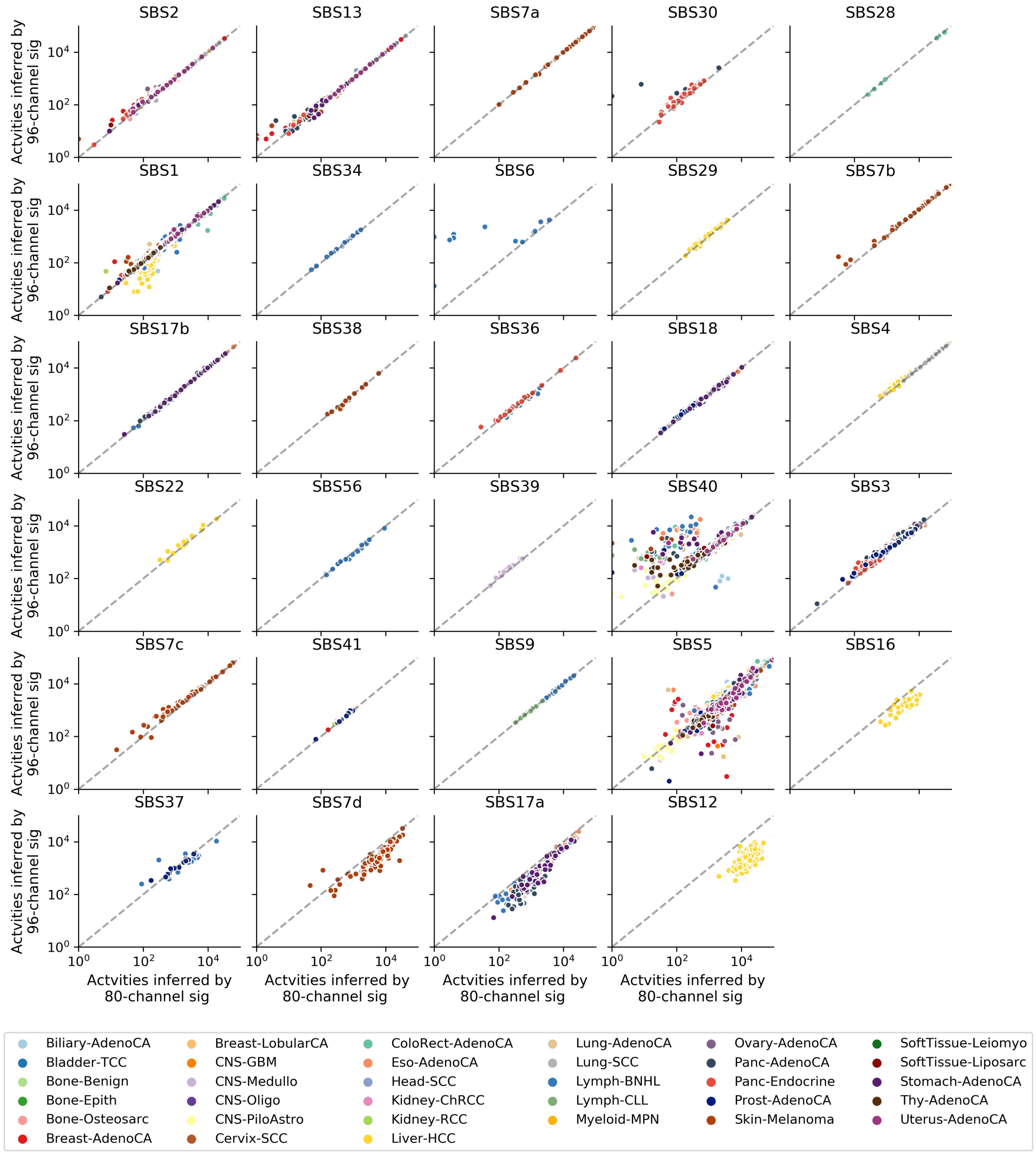

Supplementary Fig. 16 Comparison of refitted signature activities of 80-channel and 96-channel spectrum in PCAWG data.

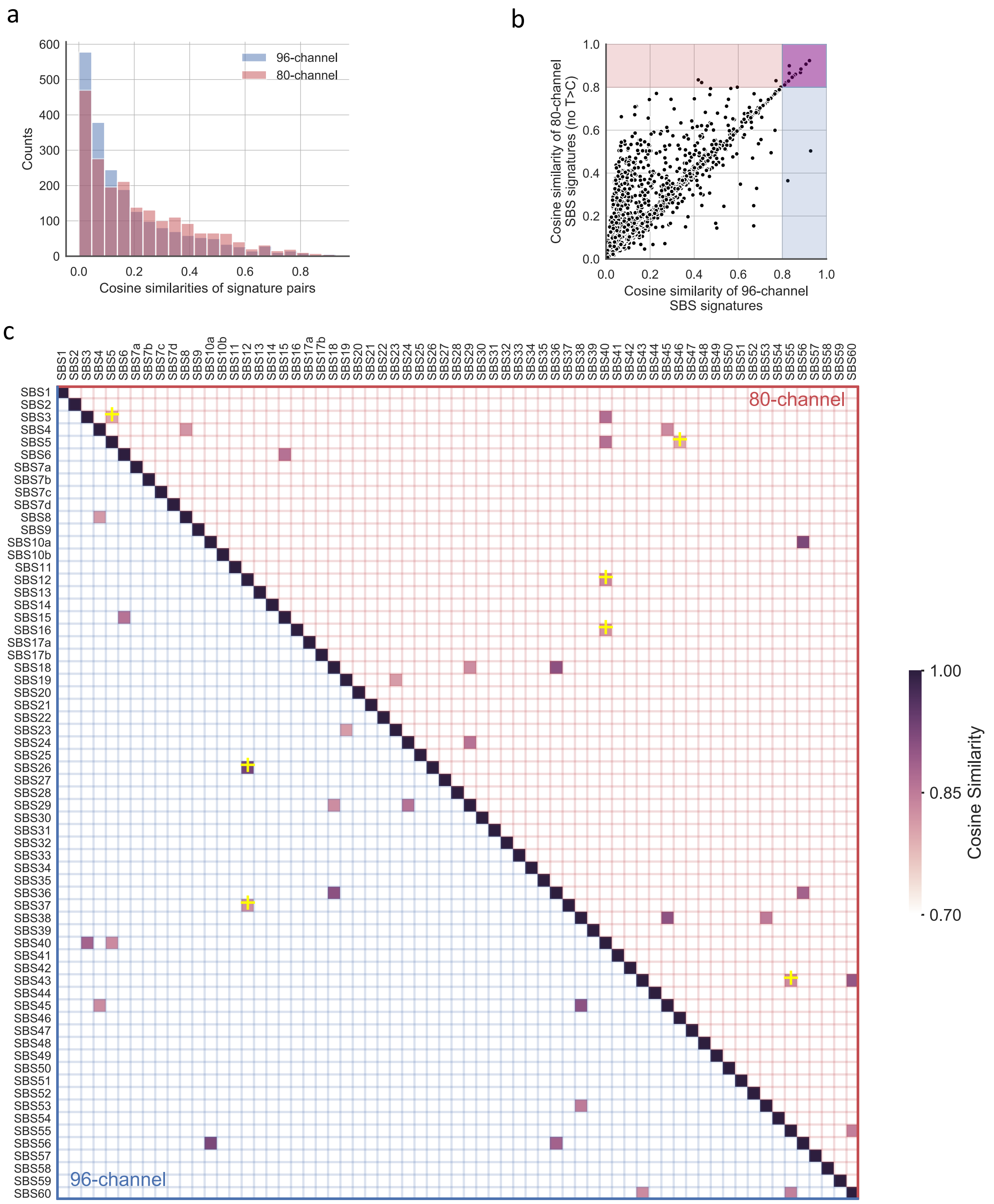

Supplementary Fig. 17 Comparison of signature similarities using 96-channel and 80-channel (without T>C) spectrum. a, Histogram of cosine similarities for signature pairs using 96-channel (96c; blue) and 80-channel (80c; pink). **b**, Scatter plot of pair wise cosine similarities using 96c and 80c signatures. Highly similar (>0.8) signature pairs are highlighted in the plot: 1) purple area shows signature pairs that are highly similar in both signature settings (96c and 80c); 2) blue area contains signature pairs are highly similar by using 96c profiles, but not highly similar by using 80c; and 3) pink area shows pairs with high similarity by using 80c not 96c. **c**, Highly similar signature pairs using 96c and/or 80c. The upper and lower triangles show the signature pairs calculated using 80c and 96c, respectively. The signature pair with + symbol represents it only exists by using 80c or by using 96c. The pairs with + symbol in the upper triangle are the dots from the pink area in (**b**), and those in the lower triangle are from blue area in (**b**).

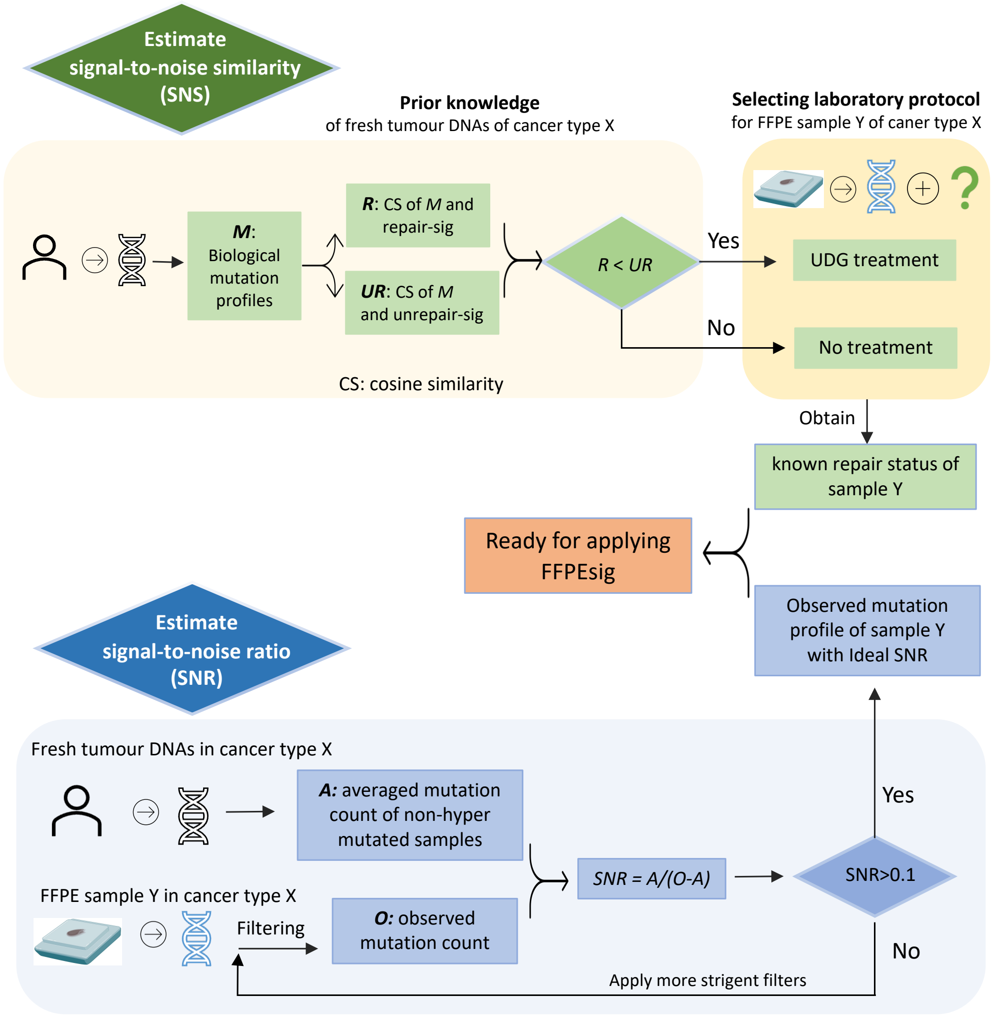

Supplementary Fig. 18 Recommended workflow for applying FFPEsig in FFPE samples. Our analysis identified two main factors that can affect noise correction accuracy of FFPEsig:1) signal-to-noise similarity (SNS), and 2) signal-to-noise ratio (SNR). Here, we demonstrate how to implement this knowledge to improve FFPEsig performance for real FFPE samples. Firstly, we recommend the potential users to leverage *prior* knowledge from available fresh tumours whether a targeted cancer type *X* has more similar biological mutation patterns to the unrepaired (*UR*) or repaired (*R*) FFPE signatures (shown in the light-yellow shaded box). For example, if the general biological signal is more similar to the unrepaired signature, we suggest the potential users to repair the FFPE DNA (from sample *Y*) using UDG treatment. Thus, we can obtain an ideal treatment status for a sample *Y* in cancer type *X*. Secondly, we suggest the potential users to compute an approximate SNR for sample *Y*. Estimation of SNR can be obtained via *A*/(*O*-*A*), where *O* is the total number of observed FFPE mutations in FFPE sample *Y* and *A* is the averaged mutation load of the non-hyper mutated tumours in the cancer type *X* (e.g., mutation load<5x10^4). Because FFPEsig performs well on hyper-mutated tumours regardless of the noise level (Fig. 2g-h), and they can be identified without FFPE artefacts being removed due to their very high and distinctive mutation patterns, or via orthogonal methods. Parts of the figure were drawn by using pictures from Servier Medical Art. Servier, which is licensed under a Creative Commons Attribution 3.0 Unported License (https://creativecommons.org/licenses/by/3.0/).

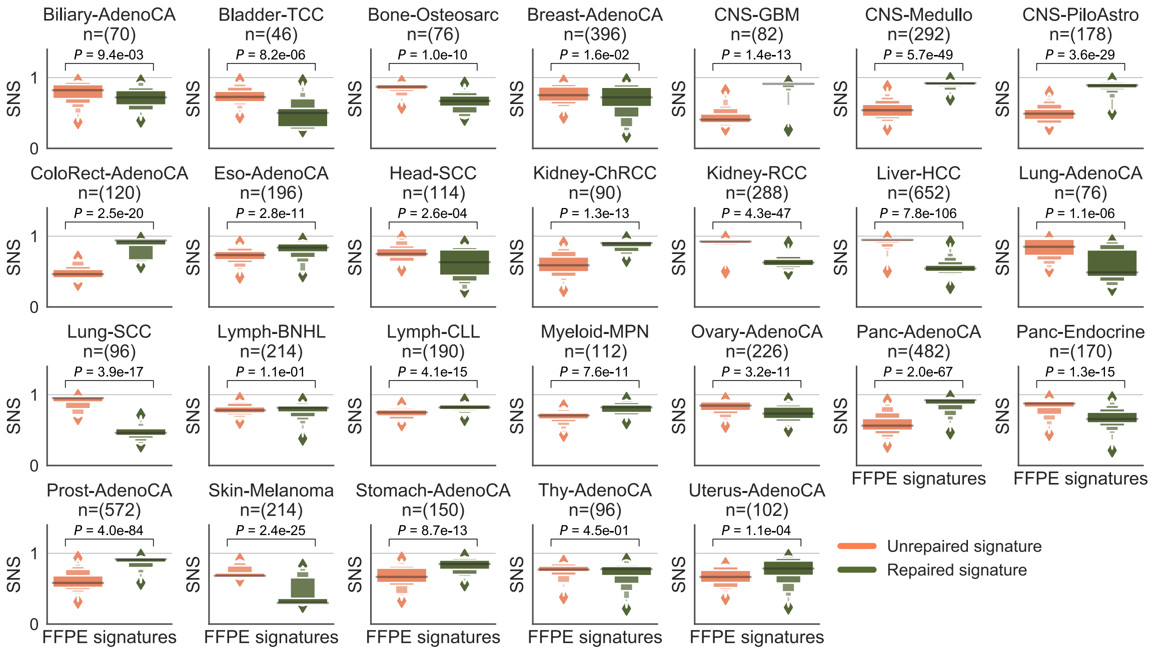

Supplementary Fig. 19 Cosine similarities between FFPE signatures and biological mutation profiles in fresh tumours from PCAWG project. We grouped PCAWG samples to multi-cancer types (showing those with sample size >20) in the figure. For each cancer type, we calculated the cosine similarities between biological mutational patterns observed in fresh tumours (the signal) and the two FFPE noise patterns discovered in this study (the noise). The y-axis shows the results, termed as SNS, which refers to signal-to-noise similarity. The x-axis shows separate groups: repaired noise pattern (olive-green coloured box) or unrepaired noise pattern (coral coloured box). Data are presented using a Letter-Value plot and the black line in the middle box corresponds to the median of the dataset. Every further step splits the remaining data further into two halves so that we get the fourths, eights etc.. The significant difference for each subgroup is measured by two-sided Mann-Whitney *U* test. We recommend the potential users of FFPEsig to use this prior knowledge while deciding whether the chemical treatment should be applied in the experimental protocols (see Supplementary Fig. 18). For example, in lung-SCC, we recommend to use UDG treatment in DNA extraction as the true biological signal of this cancer type is highly similar to the unrepaired-FFPE signature, whereas no chemical repair treatment should be applied to colorectal cancers.

References

1. Flores Bueso, Y., Walker, S. P. & Tangney, M. Characterization of FFPE-induced bacterial DNA damage and development of a repair method. *Biol Methods Protoc* **5**, bpaa015 (2020).
2. Chen, G., Mosier, S., Gocke, C. D., Lin, M. T. & Eshleman, J. R. Cytosine Deamination Is a Major Cause of Baseline Noise in Next-Generation Sequencing. *Mol. Diagn. Ther.* **18**, 587–593 (2014).
3. Williams, C. *et al.* A high frequency of sequence alterations is due to formalin fixation of archival specimens. *Am. J. Pathol.* **155**, 1467–1471 (1999).
4. Do, H., Wong, S. Q., Li, J. & Dobrovic, A. Reducing sequence artifacts in amplicon-based massively parallel sequencing of formalin-fixed paraffin-embedded DNA by enzymatic depletion of uracil-containing templates. *Clin. Chem.* **59**, 1376–1383 (2013).
5. Do, H. & Dobrovic, A. Dramatic reduction of sequence artefacts from DNA isolated from formalin-fixed cancer biopsies by treatment with uracil- DNA glycosylase. *Oncotarget* **3**, 546–558 (2012).
6. Yost, S. E. *et al.* Identification of high-confidence somatic mutations in whole genome sequence of formalin-fixed breast cancer specimens. *Nucleic Acids Res.* **40**, e107 (2012).
7. Spencer, D. H. *et al.* Comparison of clinical targeted next-generation sequence data from formalin-fixed and fresh-frozen tissue specimens. *J. Mol. Diagn.* **15**, 623–633 (2013).
8. Oh, E. *et al.* Comparison of accuracy of whole-exome sequencing with formalin-fixed paraffin-embedded and fresh frozen tissue samples. *PLoS One* **10**, 1–13 (2015).
9. Serizawa, M. *et al.* The efficacy of uracil DNA glycosylase pretreatment in amplicon-based massively parallel sequencing with DNA extracted from archived formalin-fixed paraffin-embedded esophageal cancer tissues. *Cancer Genet.* **208**, 415–427 (2015).
10. Lin, M.-T. *et al.* Clinical validation of KRAS, BRAF, and EGFR mutation detection using next-generation sequencing. *Am. J. Clin. Pathol.* **141**, 856–866 (2014).
11. Ofner, R. *et al.* Non-reproducible sequence artifacts in FFPE tissue: an experience report. *J. Cancer Res. Clin. Oncol.* **143**, 1199–1207 (2017).
12. Gallegos Ruiz, M. I. *et al.* EGFR and K-ras mutation analysis in non-small cell lung cancer: Comparison of paraffin embedded versus frozen specimens. *Cell. Oncol.* **29**, 257–264 (2007).
13. Sah, S. *et al.* Functional DNA quantification guides accurate next-generation sequencing mutation detection in formalin-fixed, paraffin-embedded tumor biopsies. *Genome Med.* **5**, 77 (2013).
14. Alborelli, I. *et al.* Robust assessment of tumor mutational burden in cytological specimens from lung cancer patients. *Lung Cancer* **149**, 84–89 (2020).
15. Parker, J. D. K. *et al.* Fixation Effects on Variant Calling in a Clinical Resequencing Panel. *J. Mol. Diagn.* **21**, 705–717 (2019).
16. Quach, N., Goodman, M. F. & Shibata, D. In vitro mutation artifacts after formalin fixation and error prone translesion synthesis during PCR. *BMC Clin. Pathol.* **4**, 1 (2004).
17. Marchetti, A., Felicioni, L. & Buttitta, F. Assessing EGFR mutations. *The New England journal of*

medicine vol. 354 526–8; author reply 526–8 (2006).

18. Wong, C., DiCioccio, R. A., Allen, H. J., Werness, B. A. & Piver, M. S. Mutations in BRCA1 from fixed, paraffin-embedded tissue can be artifacts of preservation. *Cancer Genet. Cytogenet.* **107**, 21–27 (1998).
19. Do, H. & Dobrovic, A. Limited copy number-high resolution melting (LCN-HRM) enables the detection and identification by sequencing of low level mutations in cancer biopsies. *Mol. Cancer* **8**, 82 (2009).
20. Lamy, A. *et al.* Metastatic colorectal cancer KRAS genotyping in routine practice: results and pitfalls. *Mod. Pathol.* **24**, 1090–1100 (2011).
21. Prentice, L. M. et al. Formalin fixation increases deamination mutation signature but should not lead to false positive mutations in clinical practice. *PLoS One* **13**, 94080 (2018).
22. Bhagwate, A. V. et al. Bioinformatics and DNA-extraction strategies to reliably detect genetic variants from FFPE breast tissue samples. *BMC Genomics* **20**, 1–10 (2019).
23. Van Allen, E. M. et al. Whole-exome sequencing and clinical interpretation of formalin-fixed, paraffin-embedded tumor samples to guide precision cancer medicine. *Nat. Med.* **20**, 682–688 (2014).
24. Alexandrov, L. B. et al. The repertoire of mutational signatures in human cancer. *Nature* **578**, 94–101 (2020).
25. ICGC/TCGA Pan-Cancer Analysis of Whole Genomes Consortium. Pan-cancer analysis of whole genomes. *Nature* **578**, 82–93 (2020).
