# Supplementary material for: The mutational signatures of formalin fixation on the human genome
Source: Nat Commun. 2022 Sep 6;13:4487. doi: 10.1038/s41467-022-32041-5 (PMC9448750; doi:10.1038/s41467-022-32041-5)
Supplement: Supplementary file 3 — Description to Additional Supplementary Information [file 41467_2022_32041_MOESM3_ESM.pdf]

Description of Additional Supplementary Files

Supplementary Data 1 FFPE artefacts signatures in 96-channel format

Supplementary Data 2 Mutational profiles of FFPE CRC samples

Supplementary Data 3 Applying recommended FFPEsig workflow on simulated FFPE samples
