# Supplementary material for: The mutational signatures of formalin fixation on the human genome
Source: Nat Commun. 2022 Sep 6;13:4487. doi: 10.1038/s41467-022-32041-5 (PMC9448750; doi:10.1038/s41467-022-32041-5)
Supplement: Supplementary file 7 — Reporting Summary [file 41467_2022_32041_MOESM7_ESM.pdf]

nature research

Corresponding author(s): Trevor Graham
Ville Mustonen

Last updated by author(s): Jul 8, 2022

Reporting Summary

Nature Research wishes to improve the reproducibility of the work that we publish. This form provides structure for consistency and transparency in reporting. For further information on Nature Research policies, see our Editorial Policies and the Editorial Policy Checklist.

Statistics

For all statistical analyses, confirm that the following items are present in the figure legend, table legend, main text, or Methods section.

n/a	Confirmed	
☐	☒	The exact sample size (*n*) for each experimental group/condition, given as a discrete number and unit of measurement
☐	☒	A statement on whether measurements were taken from distinct samples or whether the same sample was measured repeatedly
☐	☒	The statistical test(s) used AND whether they are one- or two-sided<br>*Only common tests should be described solely by name; describe more complex techniques in the Methods section.*
☐	☒	A description of all covariates tested
☐	☒	A description of any assumptions or corrections, such as tests of normality and adjustment for multiple comparisons
☐	☒	A full description of the statistical parameters including central tendency (e.g. means) or other basic estimates (e.g. regression coefficient) AND variation (e.g. standard deviation) or associated estimates of uncertainty (e.g. confidence intervals)
☐	☒	For null hypothesis testing, the test statistic (e.g. *F*, *t*, *r*) with confidence intervals, effect sizes, degrees of freedom and *P* value noted<br>*Give P values as exact values whenever suitable.*
☒	☐	For Bayesian analysis, information on the choice of priors and Markov chain Monte Carlo settings
☒	☐	For hierarchical and complex designs, identification of the appropriate level for tests and full reporting of outcomes
☒	☐	Estimates of effect sizes (e.g. Cohen's *d*, Pearson's *r*), indicating how they were calculated

Our web collection on statistics for biologists contains articles on many of the points above.

Software and code

Policy information about availability of computer code

Data collection: No software was used to collect the data.

Data analysis: FFPEsig is an open-source and free tool. FFPEsig (v1.0), data analysis and simulation notebooks used for generating all figures and tables are also available from https://github.com/QingliGuo/FFPEsig.

The following open-source software was used in the bioinformatic analysis:
SigProfilerMatrixGenerator (https://github.com/AlexandrovLab/SigProfilerMatrixGenerator, v1.1);
FastQC (https://www.bioinformatics.babraham.ac.uk/projects/fastqc/, v0.11.9);
BWA-MEM (https://sourceforge.net/projects/bio-bwa/files/, v0.7.17);
GATK4 (https://github.com/broadinstitute/gatk/releases, v4.0.7.0);
Skewer (https://sourceforge.net/projects/skewer/, v0.2.2);
Platypus (https://www.well.ox.ac.uk/research/research-groups/lunter-group/lunter-group/platypus-a-haplotype-based-variant-caller-for-next-generation-sequence-data, v0.8.1.1);
VCFtools(https://vcftools.github.io/downloads.html, v0.1.16);
ANNOVAR (https://annovar.openbioinformatics.org/en/latest/user-guide/download/, Version: Date: 2018-04-16 00:48:00 -0400 (Mon, 16 Apr 2018)).

For manuscripts utilizing custom algorithms or software that are central to the research but not yet described in published literature, software must be made available to editors and reviewers. We strongly encourage code deposition in a community repository (e.g. GitHub). See the Nature Research guidelines for submitting code & software for further information.

Data

Policy information about availability of data

All manuscripts must include a data availability statement. This statement should provide the following information, where applicable:

- Accession codes, unique identifiers, or web links for publicly available datasets
- A list of figures that have associated raw data
- A description of any restrictions on data availability

The BAM and the comprehensive version of VCF files for WGS FFPE-CRC data generated in this study have been deposited in the EGA database under accession code EGAS00001005331 [https://ega-archive.org/studies/EGAS00001005331]. The raw BAM data are protected and are not available due to data privacy laws, access can be obtained with the agreement with our Data Sharing Policy [https://ega-archive.org/dacs/EGAC00001002136]. Source data for Fig. 4 are available to download from our GitHub repository [https://github.com/QingliGuo/FFPEsig/blob/main/Source_Data.zip]. All raw and processed data used in our study is available to download from [https://github.com/QingliGuo/FFPEsig/tree/main/Data].

Mutations of targeted sequencing data in study 1 are available in [https://journals.plos.org/plosone/article?id=10.1371/journal.pone.0196434]. Mutations from study 2 are available upon request to the authors of the original study. Mutation from study 3 are free to download from [https://www.ncbi.nlm.nih.gov/pmc/articles/PMC4048335/]. FASTA sequences of targeted region can be downloaded from [https://www.ncbi.nlm.nih.gov/sites/batchentrez] (for study 1) and [https://m.ensembl.org/info/website/tutorials/grch37.html] (for study 2). The whole genome context mutation opportunities can be downloaded from [https://github.com/andrej-fischer/EMu].

Human genome assembly GRCh38 is downloaded from [ftp://ftp.1000genomes.ebi.ac.uk/vol1/ftp/technical/reference/GRCh38_reference_genome/GRCh38_full_analysis_set_plus_decoy_hla.fa]. PCAWG signatures, mutational profiles and signature activity data are available from [https://www.synapse.org/#!Synapse:syn11801889].

Field-specific reporting

Please select the one below that is the best fit for your research. If you are not sure, read the appropriate sections before making your selection.

☒ Life sciences ☐ Behavioural & social sciences ☐ Ecological, evolutionary & environmental sciences

For a reference copy of the document with all sections, see nature.com/documents/nr-reporting-summary-flat.pdf

Life sciences study design

All studies must disclose on these points even when the disclosure is negative.

Sample size	No samples-size calculation was carried out. We analyzed all suitable data according our exclusion criteria below. Statistical power of our analysis was performed throughout the study on all suitable data.
Data exclusions	We used the original sample sizes from cancer types without excluding any individuals. No statistical method was used to predetermine sample size. To derive statistics, we focused on cancer types or signature groups with sample size over 20.<br><br>To derive FFPE-artefact, we excluded mutations with >0.9 posterior probability of being somatic variants in study 1. To demonstrate the impact of signature decomposition results using corrected and uncorrected FFPE profiles, we focused on well-corrected samples (accuracy > 0.90). We also excluded T>C mutations in our analysis for the reason that they are more likely from other sequencing error resources, rather than formalin exposure based on our analysis.
Replication	No primary experiments were carried out. No biological or technical replicates were considered: only a single sample was analysed for each PCAWG cancer.
Randomization	Randomization is not applicable as no experimental groups included in this study.
Blinding	Blinding is not applicable as no experimental groups included in this study.

Reporting for specific materials, systems and methods

We require information from authors about some types of materials, experimental systems and methods used in many studies. Here, indicate whether each material, system or method listed is relevant to your study. If you are not sure if a list item applies to your research, read the appropriate section before selecting a response.

Materials & experimental systems

n/a	Involved in the study
☒	☐ Antibodies
☒	☐ Eukaryotic cell lines
☒	☐ Palaeontology and archaeology
☒	☐ Animals and other organisms
☐	☒ Human research participants
☒	☐ Clinical data
☒	☐ Dual use research of concern

Methods

n/a	Involved in the study
☒	☐ ChIP-seq
☒	☐ Flow cytometry
☒	☐ MRI-based neuroimaging

Human research participants

Policy information about studies involving human research participants

Population characteristics	The male patient with ulcerative colitis was diagnosed with cancer in the transverse colon at age 48 in St. Mark's Hospital, London, United Kingdom.
Recruitment	The samples were collected from St. Mark's Hospital, London, United Kingdom, followed by local protocols
Ethics oversight	Our research complies with all relevant ethical regulations. The archival FFPE samples were analysed in accordance with ethical approval from the UK Research Ethics Committee (REC: 18/LO/2051 IRAS:249008) whereby anonymised archival FFPE blocks were provided to the researchers without the requirement for patient consent.

Note that full information on the approval of the study protocol must also be provided in the manuscript.
